# Developmental switch from morphological replication to compensatory growth for salamander lung regeneration

**DOI:** 10.1111/cpr.13369

**Published:** 2022-12-04

**Authors:** Binxu Yin, Kun Zhang, Xinge Du, Hao Cai, Tingting Ye, Heng Wang

**Affiliations:** ^1^ College of Animal Science and Technology Shandong Agricultural University Taian China; ^2^ Department of Respiratory and Critical Care Medicine, People's Hospital of China Three Gorges University The First People's Hospital of Yichang Yichang China; ^3^ College of Animal Science and Technology Huazhong Agricultural University Wuhan China

## Abstract

Salamanders possess a pair of lungs for active air breathing, but the lung respiration is fully operational only during the late stage of development, particularly after metamorphosis. Larval salamanders mainly exchange air through the gills and skin, thus sparing the developing lungs. Salamanders can repair their lungs after injury, but a comparative analysis of regenerative responses between the lungs of young and adult animals is lacking. In this study, lung resections were performed in both larval and adult newts (*Pleurodeles waltl*). The cellular dynamics, tissue morphology and organ function during lung regeneration were examined and the Yap mutants were produced with CRISPR tools. We found that salamander switches the regenerative strategies from morphological replication through the blastema formation to compensatory growth via resident epithelial cells proliferation upon pulmonary resection injury as it transitions beyond metamorphosis. The larval animals achieve lung regeneration by forming a transient blastema‐like structure and regrowing full‐sized developing lungs, albeit unventilated. The adults repair injured lungs via massive proliferating epithelial cells and by expanding the existing alveolar epithelium without neo‐alveolarization. Yap signalling promotes epithelial cell proliferation and prevents epithelial‐to‐mesenchymal transition to restore functional respiration. The salamanders have evolved distinct regenerative strategies for lung repair during different phases of life. Our results demonstrate a novel strategy for functional lung recovery by inducing epithelial cell proliferation to strengthen the remaining alveoli without rebuilding new alveoli.

## Graphical abstract

Larval newts repair lungs via blastema formation and morphological replication, but adult newts restore the function of the lungs by compensatory growth. The robust activation and proliferation of alveolar epithelial cells occur, but without epithelial‐to‐mesenchymal transition (EMT). Yap promotes epithelial cell proliferation and blocks EMT and fibrosis.

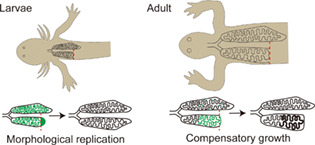

## 1 INTRODUCTION

The salamander is a unique animal model for studying tissue development and regeneration.^1^ Many of its organs have perfect regeneration capabilities and have been extensively studied, such as the limb,^2^, ^3^ brain,^4^ heart,^5^, ^6^ spinal cord,^7^ and lens.^8^, ^9^ Most of the regeneration processes occur via the formation of a blastema‐like structure and the subsequent regrowth of a morphological replica of the lost tissue, which is also called epimorphic regeneration.^10^ For instance, upon limb amputation, the wound stump is first covered by migrating epithelial cells to form a wound epidermis. Then the adult stem cells activate and mature somatic cells de‐differentiate to produce a pool of proliferating progenitor cells to construct the blastema, from which the new limb, which is identical to the original, grows. The blastema‐based regeneration was also observed upon resection of internal organs, such as the heart.^5^, ^11^ However, other organs also implement compensatory growth or congestion to restore normal physiological function after injury, such as hepatectomy in the liver, in which no blastema is formed and the original size of the organ is never restored.^12^ A recent report showed organ‐wide cell proliferation and compensatory regeneration after lung amputation in axolotls.^13^ It would be interesting to examine lung regeneration in the newt, which has a much more complex aquatic‐terrestrial lifestyle than the paedomorphic water‐dwelling axolotls and utilizes different cellular mechanisms for regeneration of tissues, such as limb^1^, ^2^, ^14^ and lens^15^, ^16^ tissues.

The evolution of vertebrate lungs occurred before or with the water‐to‐land transition, a process facilitated by the emergence of amphibians.^17^, ^18^ Unlike mammals, amphibian salamanders have multiple ways of obtaining oxygen throughout different stages of development. The aquatic larval salamanders rely mainly on gills and skin to absorb oxygen in the water, and after metamorphosis they transform into semi‐aquatic organisms and respirate mainly through the lungs for air exchange.^19^ One exception is the lungless salamanders, which never develop lungs as adults and conduct gas exchange entirely across the skin and buccopharyngeal.^19^, ^20^ Adaptation to open‐air breathing makes salamander lungs highly comparable to those of other higher species in terms of organ structure, cellular composition, gas exchange, and host defense. The lungs of salamanders consist of two unicameral lung sacs covered internally with different respiratory epithelial cells (pneumocytes), which share many similarities with those of mammals.^21^ For instance, salamander epithelial cells express thyroid transcription factor‐1 (TTF‐1) and surfactant protein B,^21^ which are equivalent to the mammalian pulmonary surfactant and host defense proteins, reflecting the highly conserved nature of pneumocytes in vertebrate lungs.

The outermost alveolar epithelial cells are most vulnerable to external insults upon lung injury, directly leading to impaired lung function. Therefore, pulmonary epithelium regeneration and the replenishment of epithelial cells are essential to restoring regular gas exchange and barrier function. Recent studies have shown that various signaling pathways, such as the BMP,^22^ Notch,^23^ Wnt/β‐catenin,^24^, ^25^ Yap/Taz,^26^, ^27^ and TGFβ^28^ pathways, are involved in epithelial regeneration in both alveoli and airways in mammals. In addition, epithelial‐to‐mesenchymal transition (EMT) is often observed during lung epithelial repair^29^ and leads to pulmonary fibrosis. In particular, EMT‐derived fibroblasts or myofibroblasts often contribute to the formation of fibrotic scars, which are usually quickly resolved during organ regeneration in newts.^30^ Whether the signaling pathways and cellular remodeling features are conserved during lung regeneration in newts remains unclear. On the other hand, reconstituting the normal epithelium remains a major challenge in several clinical lung diseases in humans, such as acute respiratory distress syndrome (ARDS)^31^ and chronic obstructive pulmonary disease (COPD),^32^ which often lead to persistent epithelial dysfunction. Thus, it is necessary to explore alternative and novel strategies for stimulating lung epithelial regeneration from animal models with extraordinary regenerative capacity.

In this study, we confirmed that newts could efficiently repair their lungs, but distinct regenerative strategies were utilized in the developing and adult animals. Specifically, we identified a developmental switch from the blastema‐based morphological replication to locally compensatory growth for newt lung recovery after resection injury. Furthermore, alveolar epithelial cells are the major drivers of adult lung repair, and Yap signaling is necessary for the activation of epithelial cells and the resolution of fibrosis.

## 2 MATERIALS AND METHODS

### 2.1 Genome editing and genotyping

Cas9 mRNA and gRNA were synthesized using mMESSAGE T7 ULTRA Transcription Kit (Invitrogen) and GeneArt Precision gRNA Synthesis Kit (Invitrogen), respectively. Genomic DNA was extracted with Qiagen DNAeasy kit. The sgRNA sequence is: GAGATGGCAAAGACCCCTTC. The IVT was performed according to the manufacturer's instructions. The sgRNAs concentration was adjusted to 50–100 ng/μl and sgRNAs were kept at −80°C until use.

Single‐cell fertilized eggs were obtained by either natural or induced breeding and injected according to previously published protocols with modifications.^33^ Briefly, 250 pg Cas9 RNA and 50 pg gRNA were mixed into 5 nl and injected into freshly laid single‐cell‐stage embryos. The screening of F0 mosaic animals was done by both genotyping and phenotype characterization, including immunohistochemistry of limbs/tails. The Yap^−/−^ F1 animals were produced by crossing between adult F0 animals. The PCR primers for genotyping were PL: CTAGGTGCTGTCTCTCCCG PR: GTTGTGTGGGTTTTGTGAGCA. Before surgery, larval and adult newts were anaesthetized with 0.01% and 0.2% of ethyl‐p‐aminobenzoate (Sigma‐Aldrich), respectively.

### 2.2 Animal husbandry, lung amputation, and chemical administration

Iberian ribbed newts *Pleurodeles waltl* were maintained in filtered tap water at 22–24°C under natural light cycles. The larvae animals were about 2 months old (stage 43). All adult animals used in this study were females and about 1 year old. A small incision of about 0.2–0.5 cm was made in the left abdomen about 1–3 cm above the hindlimbs. About 1/4 distal part of the left lung was removed with sterile surgical blades. The injured lung was carefully put back into the body and the abdominal wound was closed with absorbable sutures. No adverse reactions were evident in all animals after injury and there were no deaths throughout the experiment. For EdU‐labelling, animals were injected intraperitoneally with 50 mg/kg. All procedures were carried out in accordance with the Institutional Animal Care and Use Committee of Huazhong Agricultural University (ethics approval no.: 2018‐0125).

### 2.3 Assessment of length and weight of lungs, number of alveoli, and alveolar walls

Immediately after lung dissection and isolation, the lungs were placed in PBS for more than 15 min to wait for the tissue to shrink naturally to a stable state for lung length counting. The lungs were then fixed in 4% paraformaldehyde and dehydrated in 30% sucrose solution. The lungs were excised with a scalpel close to the trachea and the injured and the contralateral lungs were dried out for weight counting. The tissues were then embedded in OCT (CellPath) frozen in liquid nitrogen and sectioned at −30°C. We used longitudinal sections to measure the number of alveoli and the thickness of the alveolar wall.

### 2.4 Quantitative RT‐PCR

Approximately 2 mm of tissue was collected from the distal regions of uninjured lungs at 0 dpa (days‐post‐amputation) and injured lungs at 10 dpa, respectively. The removed tissue was placed in a petri dish poured with PBS to remove the blood cells. Total RNA was extracted by using TRIzol reagent (SimGen). The cDNA was synthesized by using NovoScript Plus All‐in‐one 1st Strand cDNA Synthesis SuperMix (Novoprotein). RT‐qPCR assay was performed by using a Hieff qPCR SYBR Green Mix (YEASEN) on BIO‐RAD CFX96 real‐time PCR system. In brief, 1 μg total RNA was subjected to reverse transcription in 20 μl final volume containing 10 mM dNTP mix and 2 M of anchored oligo‐dt. The mixture was incubated at 50°C for 15 min, 75°C for 5 min. The RT‐qPCR conditions were: initial denaturation at 95°C for 5 min, followed by 39 cycles of 95°C for 20 s, 60°C for 20 s, and 72°C for 20 s. The relative amount of Krt5, Trp63, and Igta6 mRNA was normalized to the amount of actin, respectively. The 2^−ΔΔCt^ method was used for evaluation of relative quantification of target gene expression. Primers are described below: actin‐F: TTGTGATGGATTCTGGTGATGGT, actin‐R: GAACTGAACCGACCAGCACT, Krt5‐F: CATCCAACGGGTGCGAAAAG, Krt5‐R: TCGTTCTCAGCAGCTGTACG, Trp63‐F: TTGCCGCAGTACACAAACCT, Trp63‐R: GTGTTGTACGGACTCGTGGA, Igta6‐F: TCTTGGAACCTGCACAAGGG, and Igta6‐R: CACGTTTTCGCGCTTCTCAT.

### 2.5 Oxygen consumption measurements

Oxygen consumption was measured at 22°C in ultrapure water inside a sealed box. Oxygen depletion in the air was monitored with an oxygen detector (AC8100, SMART SENSOR) and oxygen in the water was measured with a dissolved oxygen analyzer (DO850, APERA). The air and dissolved oxygen consumed by the animal over a 2‐h period were combined for each measurement and the oxygen consumption per unit of body weight per unit of time was calculated.

### 2.6 Immunofluorescence and Masson trichrome staining

Frozen sections (6–8 μm) were thawed at room temperature and fixed in 4% formaldehyde for 5 min. The sections were then blocked with 10% goat serum in 0.1% Triton‐X for 30 min at room temperature. The tissue sections were incubated with primary antibodies overnight at 4°C and the corresponding secondary antibodies were conjugated to Alexa Fluor 488 or 555 (Invitrogen) for 1 h. The sections were washed in PBS three times between different treatments. Primary antibodies used are following: anti‐E‐Cadherin (DSHB‐5D3, 1:200), anti‐Vimentin (DSHB‐40 E‐C, 1:200), and anti‐Yap (Cell Signalling Technology‐D8H1X, 1:100). EdU staining was performed by incubating the sections with 100 mmol/L Tris, 1 mmol/L CuSO_4_, 10 mmol/L fluorescent azide, and 100 mmol/L ascorbic acid for 30 min. Once all washing steps were completed, the coverslips were counterstained with 50 ng/mL 4′, 6‐diamidino‐2‐phenylindole (DAPI). A confocal fluorescence microscope was used to examine and image the sections. Formalin‐fixed tissues were embedded in OCT and 6–8 μm sections were stained with Masson's trichrome (Servicebio) reagent to analyze fibrosis. View sections and obtain photographs with Olympus BX‐53.

### 2.7 Data analysis

The animal experiments were not randomized. All quantifications and images were derived from at least three different individual animals, each quantification was counted from at least three different sections or samples. Data are presented as the mean ± standard error of the mean (SEM). Statistical differences were analyzed by Student's *t*‐test. Statistical analyses were performed using GraphPad Prism v8.0.

## 3 RESULTS

### 3.1 Larval animals repair the damaged lung by blastema‐based regeneration

**FIGURE 1:**
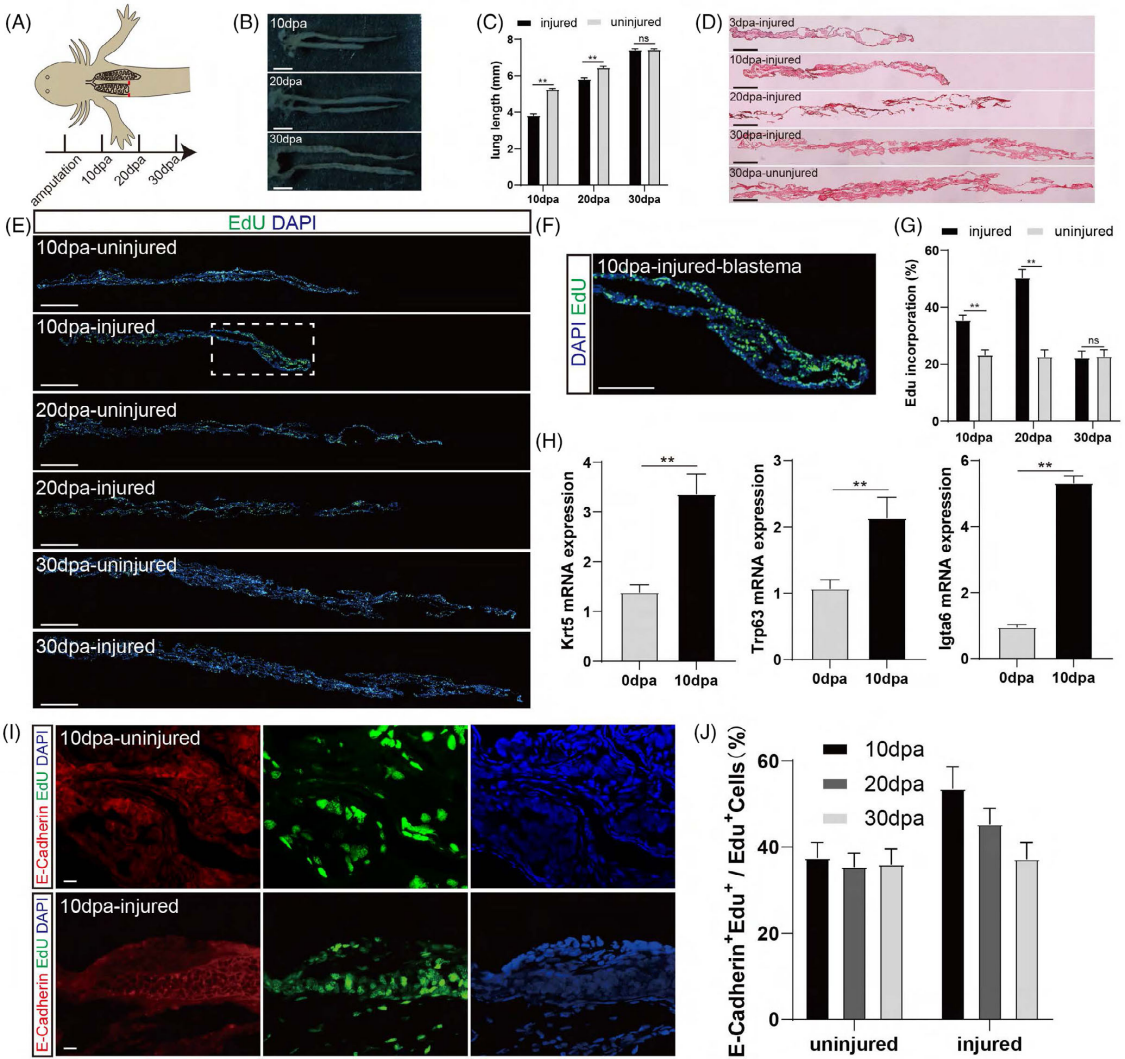
Larval newts repair the damaged lung by blastema formation to achieve morphological replication. (A) Experimental scheme. The left lung of the 2‐month‐old animal (stage 43) was amputated at 1/4 the distance from the distal tip, while the right lung was left intact. (B) Lung repair process and morphology demonstration (*n* = 6 animals/time point). Scale bar: 1 mm. (C) The lung length in (B) was measured at multiple time points over the entire regeneration period (*n* = 6). (D) Masson trichrome staining of the injured and contralateral uninjured lungs at different time points. Scale bar: 500 μm. (E) EdU staining of injured and contralateral uninjured lungs at 10, 20, and 30 dpa. EdU was administered 6 h before sample collection. Note the accumulation of EdU^+^ cells in the blastema at 10 dpa (F). Scale bar: 500 μm. (F) Demonstration of the distal region of the lung to show the blastema formation during larval lung regeneration. Scale bar: 100 μm. (G) Cell proliferation rate in (E). Data are mean ± SEM (*n* = 6). (H) The Krt5, Trp63, and Igta6 gene expression in the uninjured lung of 0 dpa and injured lung of 10 dpa. Data are mean ± SEM (*n* = 9). (I) Edu and E‐Cadherin co‐immunofluorescence staining of the injured and contralateral uninjured lungs at 10 dpa. Scale bar: 20 μm. (J) Quantification of the proliferating epithelial cells of the injured and contralateral uninjured lungs at 10, 20, and 30 dpa. Data are mean ± SEM (*n* = 3). Statistical difference was determined by Student's *t*‐test. “**” indicates *p* < 0.01. “*” indicates *p* < 0.05. “ns” indicates not significant.

We first investigated the regenerative capacity of lungs in larval newts by amputation and examined whether the regeneration occurred from the blastema or by organ‐wide proliferation similar to that in axolotls (Figure 1A). We excised approximately 1/4 of the distal part of the left lung of the 2 months old (stage 43) animal and collected the regenerating tissue at 10 dpa (days post‐amputation), 20 dpa and 30 dpa after injury. We found that the injured lung regrew gradually to reach approximately the same length as the contralateral uninjured lung at 30 dpa (Figure 1B,C), although the contralateral uninjured lung also grew. Throughout the regeneration period, we did not find any fibrosis formation, as shown by Masson trichrome staining (Figure 1D). Next, we examined the cell proliferation dynamics by the EdU assay, in which 50 mg/kg EdU was administered 6 h before tissue collection. We found that the cells in the uninjured developing lung proliferated at a relatively steady rate throughout the 30‐day‐long experimental period. The cellular proliferation rate of the damaged lung was significantly higher than that of the contralateral uninjured lung at 10 and 20 dpa and returned to comparable levels to that of the contralateral side at 30 dpa when the regeneration was nearly completed (Figure 1E–G). At 10 dpa, about half of the proliferating cells were epithelial origin as shown by co‐staining of EdU and the epithelial marker E‐cadherin, indicating that epithelial cells were actively contributing to lung regeneration (Figure 1I,J). In terms of the distribution of proliferating cells, we found an aggregation of a large number of EdU^+^ cells close to the injury site (Figure 1F). This pattern is in agreement with the typical blastema‐based regeneration and the regrown new tissue faithfully recapitulates the lost tissue. To confirm the identity of the blastema cells, we collected the blastema tissue at the distal region of the regenerating lung and identified that progenitor cell markers, such as Krt5,^34^, ^35^ Trp63,^34^, ^36^ and Igta6,^34^, ^37^ were all significantly upregulated (Figure 1H), indicating that the cellular dedifferentiation contribute to the blastema formation. Furthermore, the blastema formation and lung regeneration were completely abrogated after the blocking of cell proliferation by the administration of DNA synthesis inhibitor gemcitabine (Figure S1A,B). Thus, the larval newts can perfectly regenerate their lungs via a blastema‐like structure after resection, and we are curious to know whether adult animals can regenerate in the same way.

### 3.2 Activation and proliferation of epithelial cells in response to lung injury in adults

**FIGURE 2:**
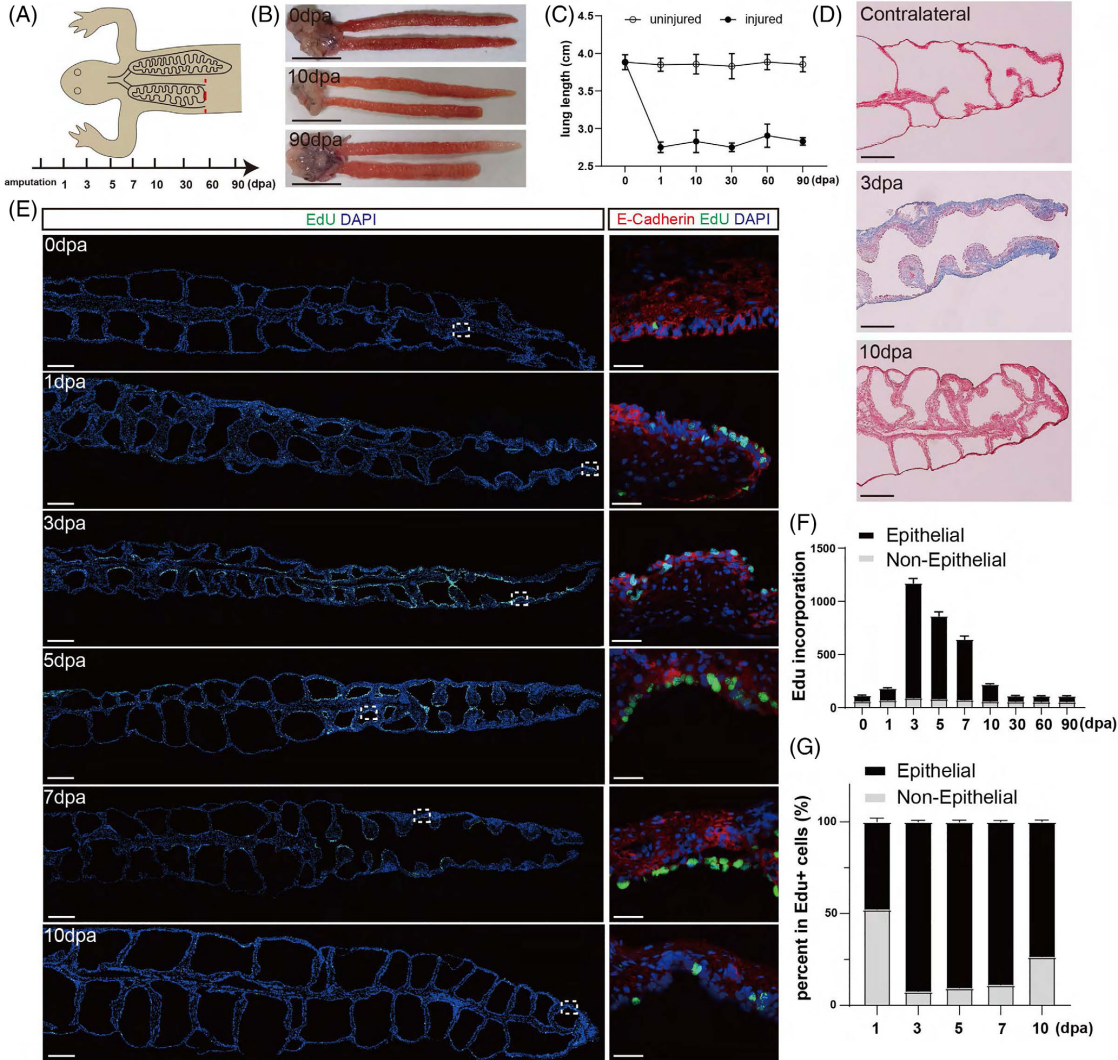
Activation and proliferation of epithelial cells in response to lung injury in the adult newt. (A) Experimental scheme. The left lung of 1‐year‐old animal was amputated at 1/4 of the distance from the distal tip, while the right lung was left intact. The samples were taken at 0, 1, 3, 5, 7, 10, 30, 60, and 90 dpa. (B) Morphology of the injured and contralateral uninjured lungs at 0, 10 and 90 dpa. Scale bar: 1 cm. (C) Quantification of the length of injured and uninjured lungs at different time points after resection. Data are mean ± SEM (*n* = 6). (D) Masson trichrome staining of the injured and contralateral uninjured lungs at different time points after amputation. Scale bar: 500 μm. (E) EdU and E‐cadherin staining of injured lungs at different time points. Scale bar: 1 mm (overview) and 100 μm (magnification). (F) Quantification of the proliferating cells and the compositions of epithelial and non‐epithelial cells in (E). Data are mean ± SEM (*n* = 4). (G) Proportions of epithelial and non‐epithelial proliferating cells. Data are mean ± SEM (*n* = 4). Statistical difference was determined by Student's *t*‐test. “**” indicates *p* < 0.01. “*” indicates *p* < 0.05. “ns” indicates not significant.

We next investigated the regenerative responses in the adult lungs by performing comparative resections in 1‐year‐old animals (Figure 2A). The amputation wounds were already healed by 10 dpa, but the morphology of the injured lung was not recovered compared to the contralateral lung during the entire course of the experimental paradigm, even at 90 dpa (Figure 2B,C). Therefore, it was evident that the adult newt lost the ability to achieve the perfect morphological replication‐based regeneration upon lung amputation. Nevertheless, we continued to examine the extent of tissue damage and cell proliferation dynamics to determine whether there was any evidence of the formation of blastema‐like structure. The Masson trichrome staining showed that, in contrast to the larval newt, the adult animals exhibited apparent fibrosis throughout the mesenchyme of the injured lung at 3 dpa (Figure 2D). It indicates that lung amputation in adults caused severe pulmonary fibrosis similar to lung injuries in other species.^38^ As injury stimulates resident cells to re‐enter the cell cycle, we examined proliferating cells at different time points by EdU assay, including 1, 3, 5, 7, 10, 30, 60, and 90 dpa, and found that proliferation peaked at 3 dpa and that proliferating cells spread throughout the whole regenerating lung (Figure 2E,F). Surprisingly, morphological identification and immunofluorescence staining suggested that the vast majority of these proliferating cells were alveolar epithelial cells, as shown by the co‐staining with EdU and E‐cadherin in both longitudinal and transverse sections (Figures 2E and S3A–C). Quantitative analysis showed that over 90% of EdU‐positive cells were of epithelial origin at 3, 5, and 7 dpa (Figure 2G). The proliferating cells returned to normal uninjured levels after 30 dpa (Figure 2F), indicating that the tissue repair was almost finished and that cell hyperplasia did not contribute to restoring the lung's original length. In addition, in the contralateral uninjured lung, no increased proliferative response was observed throughout the course of repairment of injured lung (Figure S2A,B).

### 3.3 No epithelial‐to‐mesenchymal transition during lung repair of the adult newts

**FIGURE 3:**
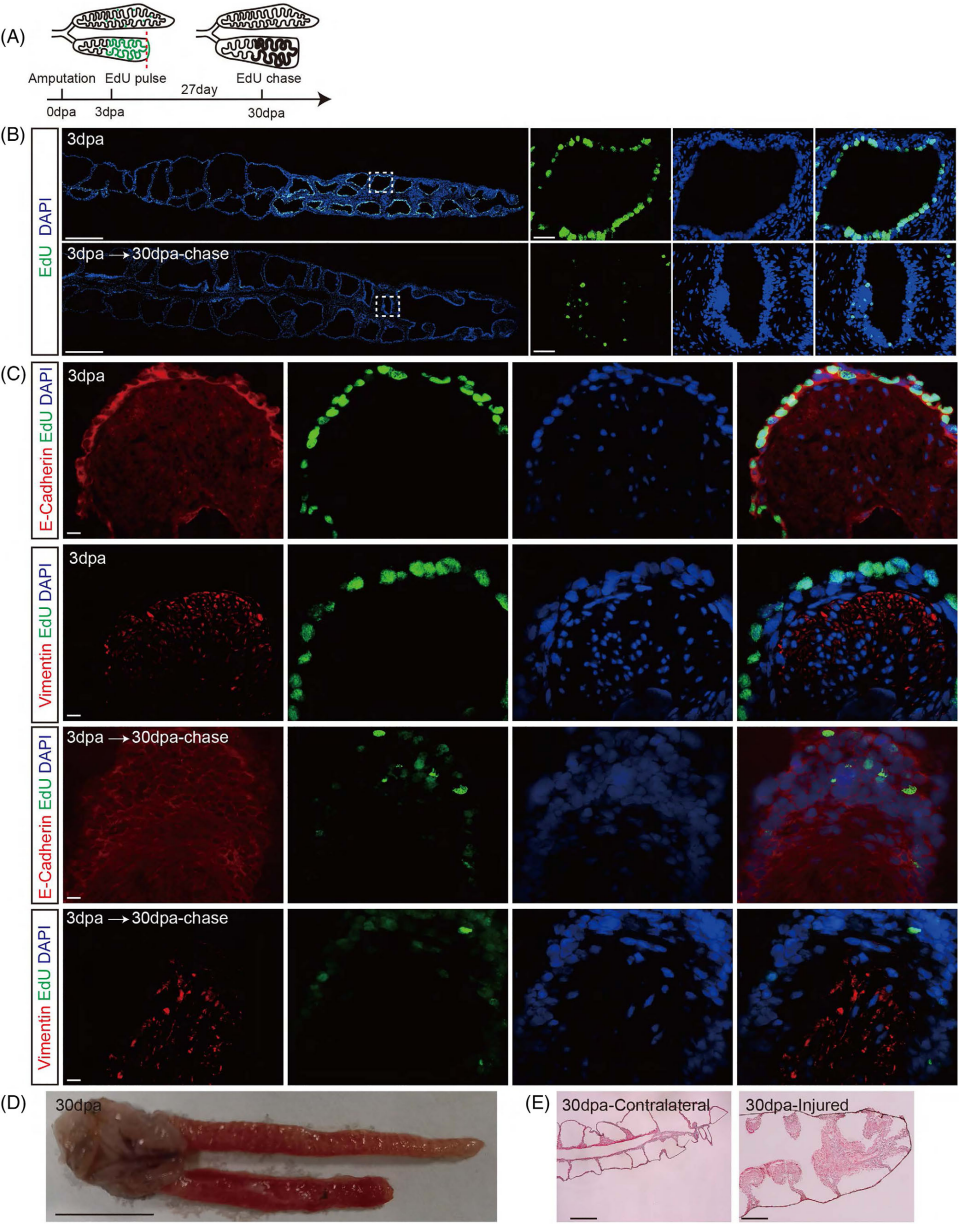
Tracking the activated epithelial cells. (A) Schematic presentation of the EdU pulse‐chase strategy. (B) Representative Edu staining at 3 dpa (pulse) and 30 dpa (chase). Scale bar: 1 mm (overview) and 100 μm (magnification). (C) Representative immunofluorescence staining of E‐cadherin (epithelial marker) and vimentin (mesenchymal marker) in the activating epithelial cells at 3 dpa and the traced cells at 30 dpa. Scale bar: 20 μm. (D) Complete anatomical images of the injured (bottom) and contralateral (top) lungs at 30 dpa. Scale bar: 1 cm. (E) Masson trichrome staining of the injured and contralateral lungs at 30 dpa. Scale bar: 500 μm.

Since the alveolar epithelial cells were the predominant cell type activated upon injury, we then tracked the fate of these cells by an EdU pulse‐chase strategy. We injected 50 mg/kg EdU into the animals at 3 dpa to label the proliferating epithelial cells and collected the lung tissues at 6 h post‐injection (6 hpi) and 30 dpa (Figure 3A). At 6 hpi, EdU incorporation was specific to epithelial cells and thus enabled us to perform long‐term tracing of these activated epithelial cells (Figure 3B). At 30 dpa, we observed very few cells with a strong EdU signal, but many faint EdU^+^ cells were also identifiable (Figure 3B). These results suggest that the proliferating epithelial cells labeled with EdU at the early regeneration stage have already undergone multiple rounds of mitotic divisions, thus diluting the EdU substance. Interestingly, both strongly and weakly, EdU^+^ cells seemed to remain in the epithelium of the alveoli, and this epithelial layer was apparently thickened during the time course of the experiment (Figure 3B).

On the other hand, the epithelial‐to‐mesenchyma (EMT) is frequently observed in wound healing and lung regeneration in mammals,^39^, ^40^ so we evaluated the contribution of activated epithelial cells to EMT by examining the expression of epithelial‐ and mesenchymal‐specific markers (E‐cadherin and vimentin, respectively). Surprisingly, we found that all the proliferating EdU^+^ cells at 3 dpa were E‐cadherin^+^/vimentin^−^, and after 27 days of regeneration, these EdU‐labeled cells were still E‐cadherin^+^/vimentin^−^ (Figure 3C). In other words, we could not detect the loss in E‐cadherin and gain in vimentin expression in the tracked EdU^+^ cells, suggesting that these activated epithelial cells did not give rise to mesenchymal lineages. Therefore, the activated alveolar epithelial cells proliferate massively but do not undergo EMT during newt lung repair. Instead, proliferating epithelial cells only contribute to the expansion of the epithelial layer within the same alveoli. Accordingly, we observed that, compared to the intact lung, the injured lung was larger in width (Figure 3D), and the alveolar wall was also thickened dramatically at 30 dpa (Figure 3E). In addition, the alveolar wall remains unchanged if the proliferation of epithelial cells was blocked by gemcitabine (Figure S4A–C), indicates that cell proliferation was necessary for the thickening of the alveolar epithelium.

### 3.4 Thickening of the epithelium of the existing alveoli is sufficient to drive functional recovery

We were intrigued by the apparent thickening of the alveolar epithelial layer during lung regeneration in the adult newt. We hypothesized that the expansion of the epithelial compartment might contribute to the functional recovery of the injured lung. Although cell proliferation returned to normal homeostasis levels at 30 dpa, functional recovery may extend beyond this time point because of physiological adaptations. Therefore, we examined lung size, weight, alveolus numbers, and alveolar wall thickness at 0, 1, 10, 30, 60, and 90 dpa.

**FIGURE 4:**
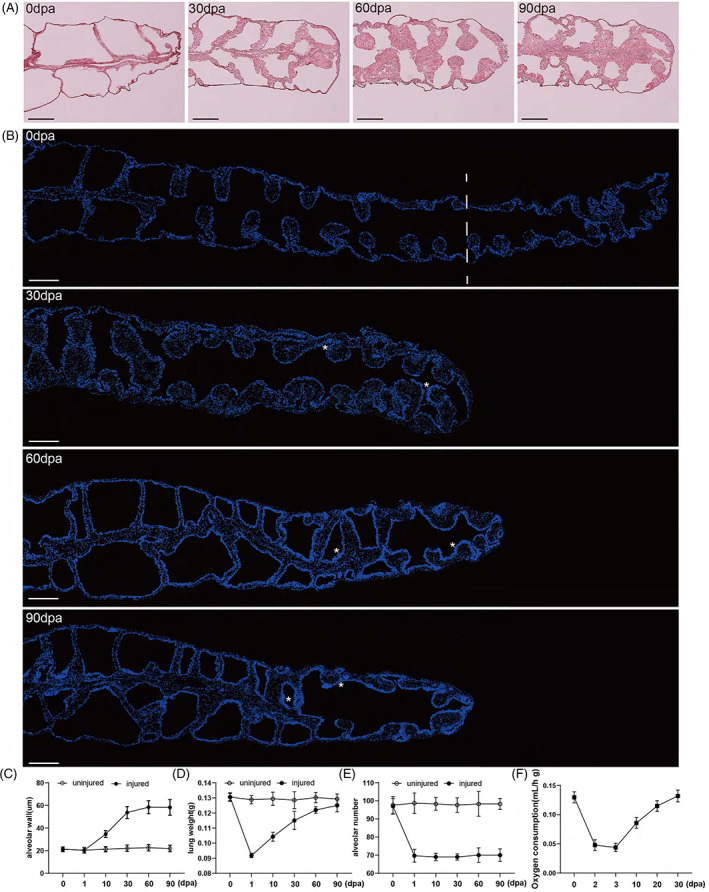
Thickening of the epithelium of the existing alveoli is sufficient to drive functional recovery in newts. (A) Masson trichrome staining of the injured lungs at different time points. Scale bar: 500 μm. (B) Representative images showing DAPI staining of the injured lungs at different time points. “*” indicates thickening of the epithelial layer. Scale bar: 500 μm. (C) Quantification of alveolar wall thickness at different time points after injury. Data are mean ± SEM (*n* = 4). (D) Weight of the regenerating lungs. Data are mean ± SEM (*n* = 4). (E) Alveolus numbers of the regenerating lungs. Data are mean ± SEM (*n* = 4). (F) Oxygen consumption levels of injured animals at different time points. Data are mean ± SEM (*n* = 3).

Macroscopically, the injured lung at 90 dpa was still much shorter than the uninjured lung, but interestingly, the injured lung was much wider than the contralateral lung and 0 dpa uninjured lung (Figure 2B,C). Both Masson trichrome staining (Figure 4A) and DAPI staining (Figure 4B) of the lung sections showed that the alveolar wall in the wounded lung gradually thickened during regeneration. From 30 dpa onward, the thickness of the alveolar epithelial layer of the remaining alveoli from the injured lung was 2‐ to 2.7‐fold that of the uninjured lung (Figure 4C). Next, we measured the weight of the lungs and found that the weight of the injured lung slowly recovered to a level comparable to that of the contralateral lung (Figure 4D), which is consistent with the widening of the lung tube in appearance. As expected, the number of alveoli in the injured lung was stable throughout the regeneration period (Figure 4E), indicating no alveologenesis. Regarding the aerobic respiration function of the lung, there was a sharp drop in oxygen consumption within 2–3 days of injury. However, the oxygen consumption levels gradually returned to normality by 30 dpa (Figure 4F), indicating the functional recovery of the injured lung. Therefore, cell proliferation‐dependent epithelialization significantly increased the thickness of the epithelial layer and alveolar wall, and improved the air‐exchange capacity of the remaining alveoli to compensate for the lost alveoli.

### 3.5 Yap signaling was required for epithelial cell proliferation and respiratory restoration

**FIGURE 5:**
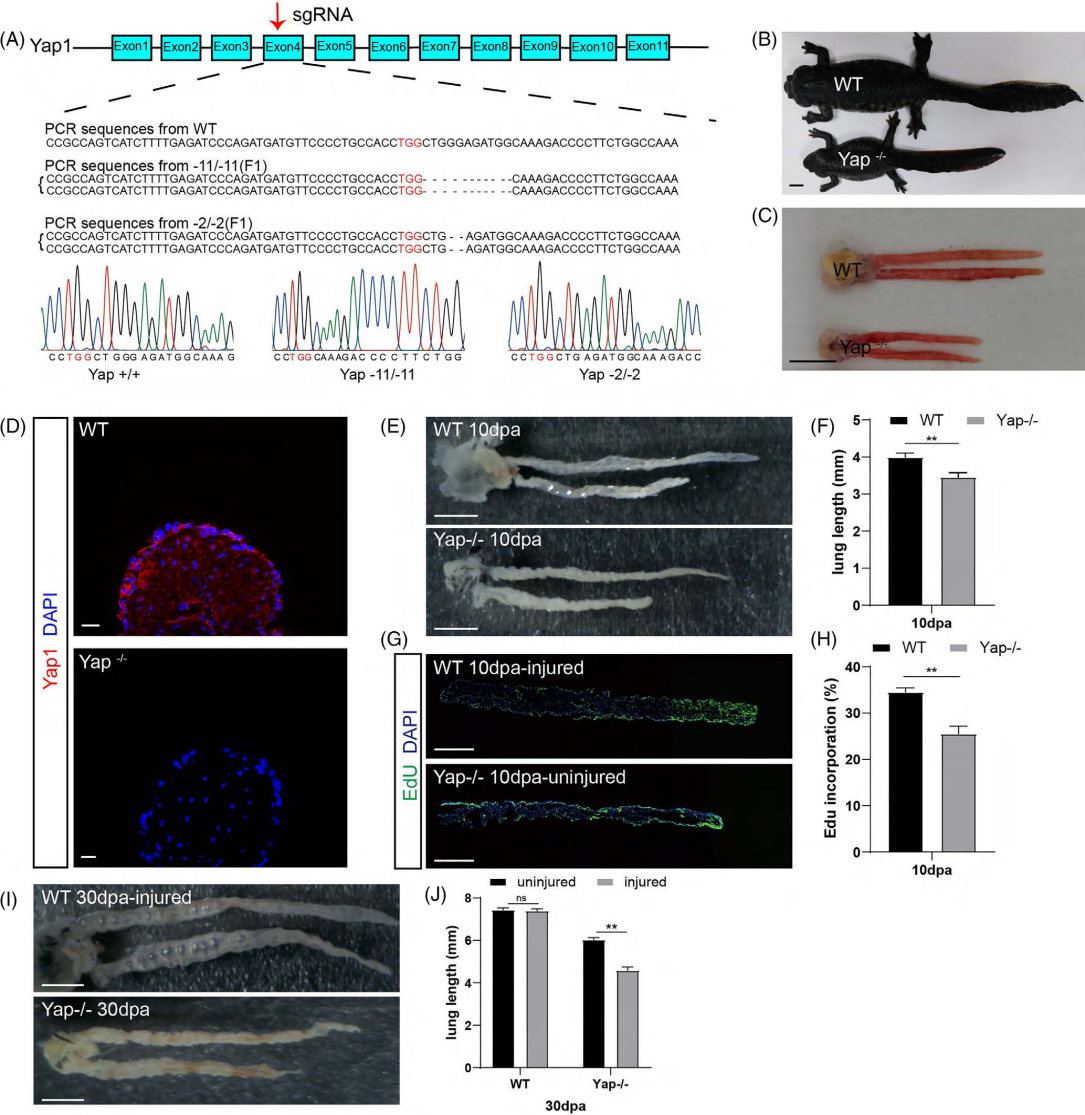
Yap knockout resulted in an overall smaller body size in newts and retarded lung regeneration in larvae. (A) Schematic representation of the gene structure and indel mutations of the Yap gene locus in the newt. (B) Body display of WT and Yap^−/−^ adult animals. Scale bar: 1 cm. (C) Anatomical view of the lungs of the WT and Yap^−/−^ animals. Scale bar: 1 cm. (D) Immunofluorescence staining showing Yap expression is lost in the lungs of Yap^−/−^ animals. Scale bar: 20 μm. (E) The morphology of lungs at 10 dpa in Yap^−/−^ and WT larvae (2‐month‐old). Scale bar: 1 mm. (F) The lung length of the regenerating lungs form Yap^−/−^ and WT larvae. Data are mean ± SEM (*n* = 3). (G) The EdU staining shows the cell proliferation in the lungs of Yap^−/−^ and WT larvae at 10 dpa. Scale bar: 500 μm. (H) The quantification of proliferating cells in the lungs of Yap^−/−^ and WT larvae at 10 dpa. Data are mean ± SEM (*n* = 3). (I) The morphology of lungs at 30 dpa in Yap^−/−^ larvae. Note that the morphology of the injured lung was not recovered to the contralateral lung at 30 dpa, whereas in the WT animals it was already fully recovered. Scale bar: 1 mm. (J) The length quantification of injured lungs and contralateral lungs at 30 dpa in WT and Yap^−/−^ larvae. Data are mean ± SEM (*n* = 3). Statistical difference was determined by Student's *t*‐test. “**” indicates *p* < 0.01. “*” indicates *p* < 0.05. “ns” indicates not significant.

The Hippo pathway and its downstream effector molecule Yap play essential roles in organ development and regeneration. Yap has been shown to be involved in alveolar stem cells proliferation and differentiation in mammalian alveolar regeneration.^26^ Considering the rapid and massive proliferation of epithelial cells during newt lung repair and the powerful pro‐proliferative function of Yap, we next wanted to determine the effect of Yap on newt lung repair. We first generated a Yap mutant line to examine the role of Yap in animal development. We employed the CRISPR/Cas9 technique to generate Yap mutants by targeting exon 4 of the Yap locus and obtained F1 homozygous knockout animals (Yap^−/−^) as shown by Sanger sequencing (Figure 5A). Immunofluorescence staining also showed that the Yap protein indeed disappeared in the lung tissues of knockout animals (Figure 5D). During development, the Yap^−/−^ F1 knockout animals were significantly smaller than the wild type (WT), with 1‐year‐old adults being only approximately 3/4 the length of the WT (Figure 5B). The length of the lung was also considerably shorter in the Yap^−/−^ animals, but the basic structure of the lungs looked similar between the Yap^−/−^ and WT animals (Figure 5C). Thus, consistent with other species,^41^ Yap signaling also controls organ size during development in newts. We next examined the lung regeneration in Yap^−/−^ larval animals. At 10 dpa, recovery of lung damage in Yap^−/−^ larvae was significantly weaker than in WT larvae (Figure 5E,F), the overall cell proliferation was significantly reduced (Figure 5H) and the blastema formation was severely delayed (Figure 5G) compare to the WT larvae. At 30 dpa, when the WT larvae already finished the regeneration to generate near identical morphology between the injured and uninjured lungs, the injured lungs in Yap^−/−^ larvae are still much shorter than the contralateral uninjured lungs (Figure 5I,J). It indicates that Yap signaling is required for cell activation and proliferation during blastema‐based lung regeneration in larval animals.

**FIGURE 6:**
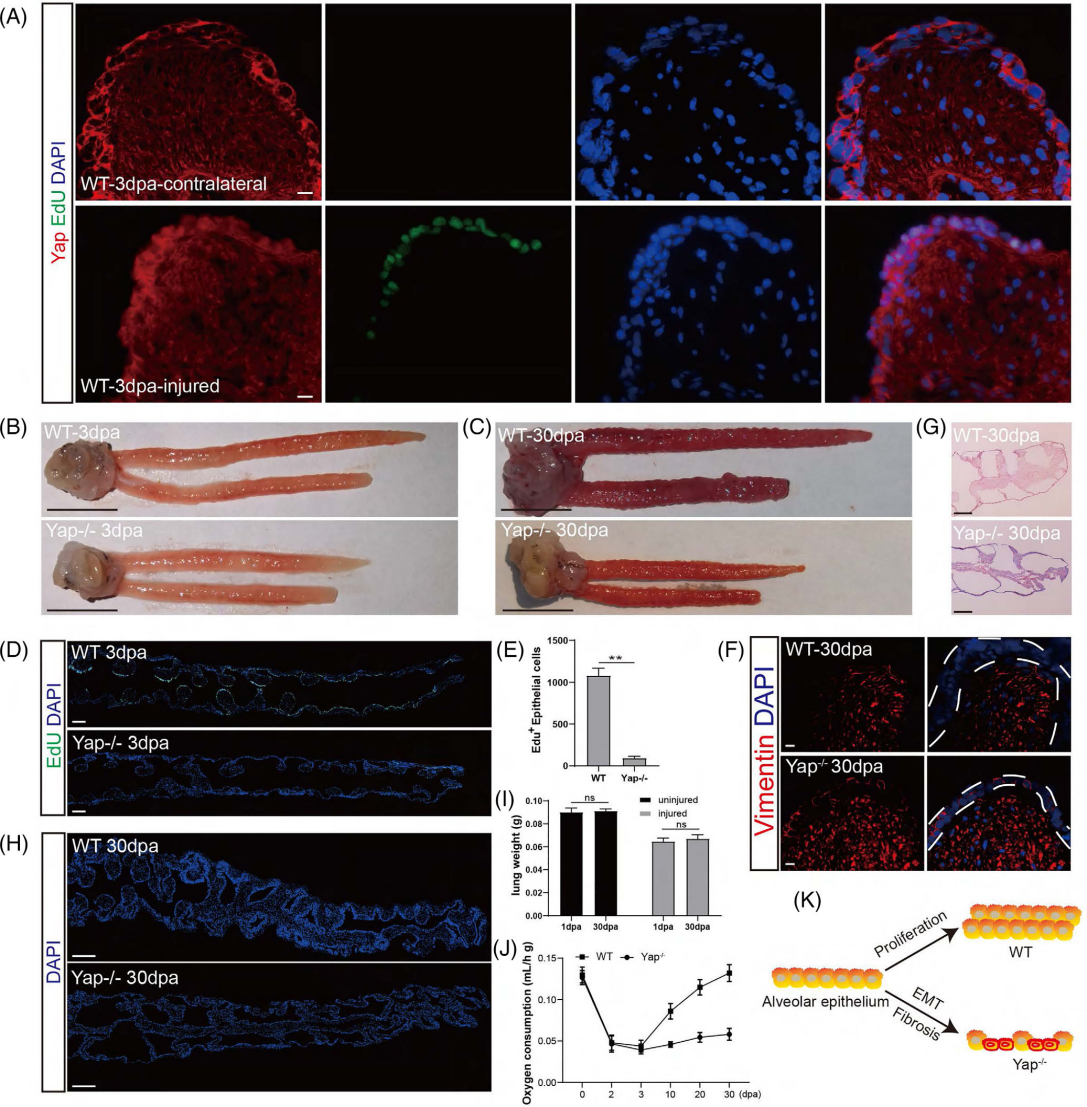
Yap deletion inhibits epithelial cell proliferation and respiratory function recovery in newts. (A) Distribution of Yap protein in uninjured and injured lungs at 3 dpa in WT animals. Scale bar: 20 μm. (B, C) Anatomical view of lungs from WT and Yap^−/−^ animals at 3 and 30 dpa. Scale bar: 1 cm. (D) Representative images showing EdU staining of regenerating lungs of WT and Yap^−/−^ animals at 3 dpa. Scale bar: 1 mm. (E) Quantification of proliferating epithelial cells at 3 dpa between WT and Yap^−/−^ animals. Data are mean ± SEM (*n* = 6). (F) Representative immunofluorescence staining of vimentin (mesenchymal marker) in the injured lungs of WT and Yap^−/−^ animals at 30 dpa. Scale bar: 20 μm. (G) Masson trichrome staining of the injured lungs of WT and Yap^−/−^ animals at 30 dpa. Scale bar: 500 μm. (H) Representative DAPI staining of lungs from WT and Yap^−/−^ animals at 30 dpa. Scale bar: 1 mm. (I) Quantification of weight of the injured and contralateral uninjured lungs at 1 and 30 dpa. Data are mean ± SEM (*n* = 3). (J) Oxygen consumption levels in WT and Yap^−/−^ animals during regeneration (*n* = 6). (K) Alternative fates of epithelial cells in Yap^−/−^ animals. Statistical difference was determined by Student's *t*‐test. “**” indicates *p* < 0.01. “*” indicates *p* < 0.05. “ns” indicates not significant.

Furthermore, we also tested the impact of Yap deletion on lung repair in adults. We first examined the involvement of Yap signaling in epithelial cell activation and proliferation in WT animals. Immunofluorescence analysis showed that in the absence of injury, Yap was predominantly expressed in the cytoplasm of the non‐cycling epithelial cells. However, 3 days after injury, many Yap^+^/EdU^+^ double‐positive cells appeared, and the Yap signal was relocated into the nuclei of the activated cells (Figure 6A). These results indicate that Yap signaling was indeed induced in the activated epithelial cells after injury. We performed lung resections on both the Yap^−/−^ and WT adult animals (Figure 6B) and examined the cell activation at 3 dpa, during which time the epithelial cells proliferated most robustly. We found that in the Yap^−/−^ animals, the proliferation of epithelial cells was almost completely lost at 3 dpa (Figure 6D,E). This is consistent with previous reports of delayed alveolar epithelial cell regeneration following Yap deletion.^27^ We then followed lung repair for a longer period of time in both WT and Yap^−/−^ animals. We found that at 30 dpa, the damaged lung was significantly larger in diameter than the contralateral uninjured lung in the WT animals, whereas in the Yap^−/−^ animals, the injured lung was not growing at all (Figure 6C). Furthermore, we carefully examined the cellular structures in the lung and found that the alveolar epithelial cells were E‐cadherin^+^ vimentin^−^ in both WT and Yap^−/−^ animals without injury (Figure S5). However, upon injury, the WT alveolar epithelial cells proliferated and remained vimentin^−^, whereas Yap^−/−^ epithelial cells did not proliferate but expressed a significant amount of vimentin (Figure 6F), indicating that these epithelial cells underwent EMT instead of proliferation upon the loss of Yap signaling. Consequently, in the injured lungs of Yap^−/−^ animals, we found persistent fibrosis even at 30 dpa (Figure 6G), which had resolved as early as 10 dpa (Figure 2D) in WT animals. Hence, the Yap^−/−^ alveolar epithelial cells failed to proliferate in response to injury. Instead, they underwent EMT and contributed to pulmonary fibrosis, hindering lung recovery.

Since epithelial cell proliferation was severely impaired in the injured lungs of Yap^−/−^ animals, the epithelial layer did not enlarge and seemed unchanged from 3 to 30 dpa (Figure 6H). Thus, the loss of Yap blocked the proliferation of epithelial cells and prevented the thickening of the epithelial layer. Accordingly, the weight of the damaged lungs of the Yap mutant did not increase from 1 to 30 dpa (Figure 6I). In addition, oxygen consumption declined in both the WT and Yap^−/−^ animals upon injury, but only the WT animals showed gradually recovery of oxygen consumption. The oxygen consumption remained low in Yap^−/−^ animals even at 30 dpa (Figure 6J). Therefore, the lack of effective replenishment of epithelial cells leads to reduced oxygen uptake and ultimately an inability to restore normal respiratory function. We found that Yap is a crucial regulator of the rapid activation and proliferation of alveolar epithelial cells in newts after lung injury and helps to restore lung function. The Yap^−/−^ epithelial cells are not activated promptly in response to injury but instead undergo EMT, eventually leading to the development of progressive pulmonary fibrosis (Figure 6K).

## 4 DISCUSSION

This study shows that after resection injury in larval newts, lung regeneration follows a typical morphological replication pattern based on blastema formation. In contrast, adult newts fail to form a regenerative blastema and can only rely on epithelial cell proliferation to enlarge the existing alveoli and restore the respiratory function of the lung. Repair after lung injury in adults is mainly dependent on the rapid activation and proliferation of epithelial cells within a week of injury, producing large numbers of progeny cells to compensate for the loss of functional epithelial cells in the lung. Hence, the newt has evolved distinct regenerative mechanisms for lung repair during different phases of life. The distinct respirational utilization of the lung at different developmental stages may result in the switch from blastema‐based regeneration to compensatory growth after pulmonary injury. It seems that the developing lungs of larval newts did not engage in air exchange at all and that the animals were not in urgent need of lung repairment for breathing upon injury. Consequently, the blastema is formed to regenerate a perfect morphological replication of the original lung for full utilization and maximized performance in the future. However, in adults, the lungs are the primary organ for constant breathing, and the acute reduction in oxygen intake upon injury may cause severe life‐threatening damage to other organs. Thus, the adult animals quickly mounted a robust activation of resident epithelial cells in response to the injury and enhanced the function of remaining alveoli to restore active breathing. We proposed that the newt could alternate regeneration strategies to restore either the morphology or function of the organ based on the severity of the injury and the priority of the impaired function. Whether this developmental switch of regenerative mechanisms also applies to other types of lung injuries or other tissue injury models require further study. Interestingly, the lung regeneration exhibits both similarities and differences between the axolotls and newts. Both species elicit a wide‐range proliferation response throughout the injured lung, but after that, the axolotls could mobilize a variety of different types of cells to recover the original morphology of the lung,^13^ whereas the adult newts mainly utilize the epithelial cells to strengthen the remnant alveoli without restoring the tissue morphology. In addition, the contralateral uninjured lung was responsive to the injury, as shown by the increased cell proliferation, in the axolotls but not in newts.

The alveolar epithelium is essential for lung gas exchange, fluid balance, and host defense. The current data combined with the cellular features from the axolotl^21^ showed many highly conserved features in the alveolar epithelial cells of salamanders compared to other species. In mammals, insufficient epithelial cell self‐renewal (4–5 weeks of turnover time)^42^ and the limited population of alveolar epithelial progenitors make the repair after acute lung injury extremely inefficient.^43^, ^44^ For instance, epithelial cells are mostly depleted in the lungs of severe COVID‐19 patients, and only 1%–10% of the remaining epithelial cells can proliferate, but they actively contribute to fibrosis, which hampers lung regeneration.^45^ In contrast, the newt alveolar epithelial cells exhibit a superior proliferative capacity than the remaining cell types in the lungs upon injury. Further analysis of the signalling factors that trigger this extraordinary proliferative response of alveolar epithelial cells would provide new clues for treating human lung diseases. Furthermore, it is also exciting that these activated epithelial cells do not undergo EMT and thus do not contribute to pulmonary fibrosis. Instead, they remain in the epithelial layer, resulting in a thicker and folded epithelium wall that could greatly expand the alveolar surface area for air exchange after the lung was fully ventilated. On the other hand, we hypothesized that the observed fibrosis during the early stage of adult lung regeneration is mainly produced by native fibroblasts. In conclusion, the explosive proliferative response in adult newt lungs upon resection injury is highly specific to the epithelial cells, and the activated epithelial cells exclusively replenish the lost cells to improve respiratory function without causing adverse effects such as fibrosis. The current study provided us with new avenues for developing regenerative therapies aiming to restore organ function without reconstituting the original morphology, even though they are capable of doing so.

Yap signaling is involved in various regenerative processes in different tissues.^46^ For example, in the lungs of mammals, Yap mediates the process of alveolar regeneration by sensing the increased mechanical tension after pneumonectomy.^26^ We observed that epithelial cells failed to proliferate after injury in adult Yap mutant newts. Since the lungs of newts are composed of two separated single‐chambered lung sacs and they may not affect each other. Thus, the injured lung on one side is transformed into a “leaky balloon” after distal resection. It is conceivable that the mechanical pressure exerted on the epithelium before and after injury must be different. Therefore, it is worth exploring further how changes in mechanical pressure could rapidly modulate Yap's nuclear translocation or expression levels and the downstream effectors that stimulate cell proliferation. Interestingly, when Yap signaling is missing, newt epithelial cells cease proliferation and adopt an alternative cell fate to undergo EMT and contribute to undesirable pulmonary fibrosis. Our data are consistent with previous studies demonstrating the importance of Yap for the proliferation and differentiation of lung epithelial progenitor cells.^26^, ^27^, ^47^, ^48^ Thus, precise activation of Yap signaling in epithelial cells may help to reduce and even reverse fibrosis.

In conclusion, we discovered a developmental stage‐specific shift in lung regeneration in the newt. The current study provides new clues for designing specific regenerative strategies that target epithelial cells for functional regeneration at the expense of high‐fidelity structural reconstitution of the lung. Instead of seeking adult stem cells to regenerate the damaged tissues or organs to their original size and morphology, efficiently stimulating and enhancing the specialized type of differentiated cells were also applicable for functional recovery.

## AUTHOR CONTRIBUTIONS

Binxu Yin and Heng Wang conceived and designed the study. Binxu Yin prepared the data. All authors analyzed the data and wrote the paper. All authors read and approved the final version of the manuscript.

## CONFLICT OF INTEREST

The authors declare no conflict of interest.

## Supporting information


**FIGURE S1.** The cell proliferation and blastema formation were inhibited after the gemcitabine (GEM) treatment in the regenerating lungs of larval salamanders
**FIGURE S2.** The contralateral uninjured lung exhibited constant low and minimal proliferation throughout the whole regeneration period
**FIGURE S3.** The identification of proliferating epithelial cells on transverse sections of 3dpa lungs
**FIGURE S4.** The cell proliferation and lung regeneration were inhibited after the gemcitabine (GEM) treatment in the adult salamanders
**FIGURE S5.** The epithelial cells in WT and Yap mutant animals are similar before injury

## Data Availability

The data supporting the conclusions of this article are included in this article and its additional files.
