# Supplementary material for: Developmental switch from morphological replication to compensatory growth for salamander lung regeneration
Source: Cell Prolif. 2022 Dec 4;56(3):e13369. doi: 10.1111/cpr.13369 (PMC9977668; doi:10.1111/cpr.13369)
Supplement: Supplementary file 1 — FIGURE S1. The cell proliferation and blastema formation were inhibited after the gemcitabine (GEM) treatment in the regenerating lungs of larval salamanders FIGURE S2. The contralateral uninjured lung exhibited constant low and minimal proliferation throughout the whole regeneration period FIGURE S3. The identification of proliferating epithelial cells on transverse sections of 3dpa lungs FIGURE S4. The cell proliferation and lung regeneration were inhibited after the gemcitabine (GEM) treatment in the adult salamanders FIGURE S5. The epithelial cells in WT and Yap mutant animals are similar before injury [file CPR-56-e13369-s001.pdf]

Supplementary Figures

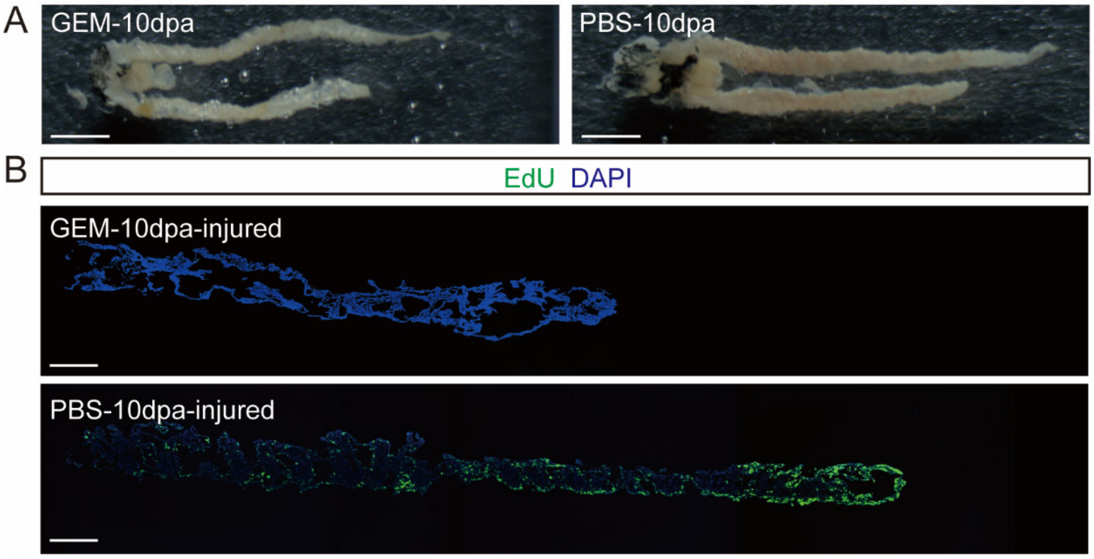

Figure S1. The cell proliferation and blastema formation were inhibited after the gemcitabine (GEM) treatment in the regenerating lungs of larval salamanders.

(A) The morphology of the lungs at 10dpa in GEM and PBS-treated animals. n=3 animals. Scale bar: 1mm.

(B) The EdU+ proliferating cells in the lungs at 10dpa in GEM and PBS-treated animals. Scale bar: 500 μm.

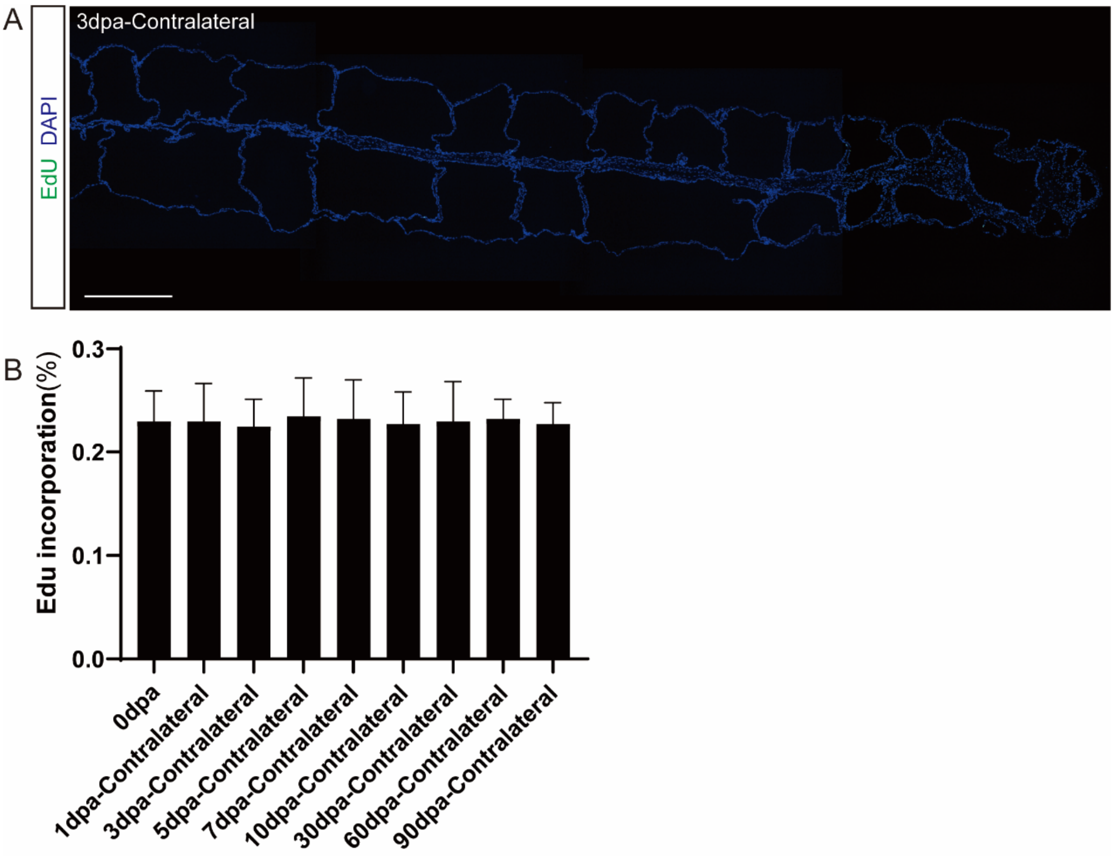

Figure S2. The contralateral uninjured lung exhibited constant low and minimal proliferation throughout the whole regeneration period.

(A) Representative pictures showing the EdU staining of the contralateral uninjured lung at 3 dpa. Note the very few EdU+ cells in the lung. Scale bar: 1 mm

(B) Quantification of EdU+ proliferating cells in the contralateral uninjured lung at different time points after injury. n=4 animals. Data are mean ± SEM.

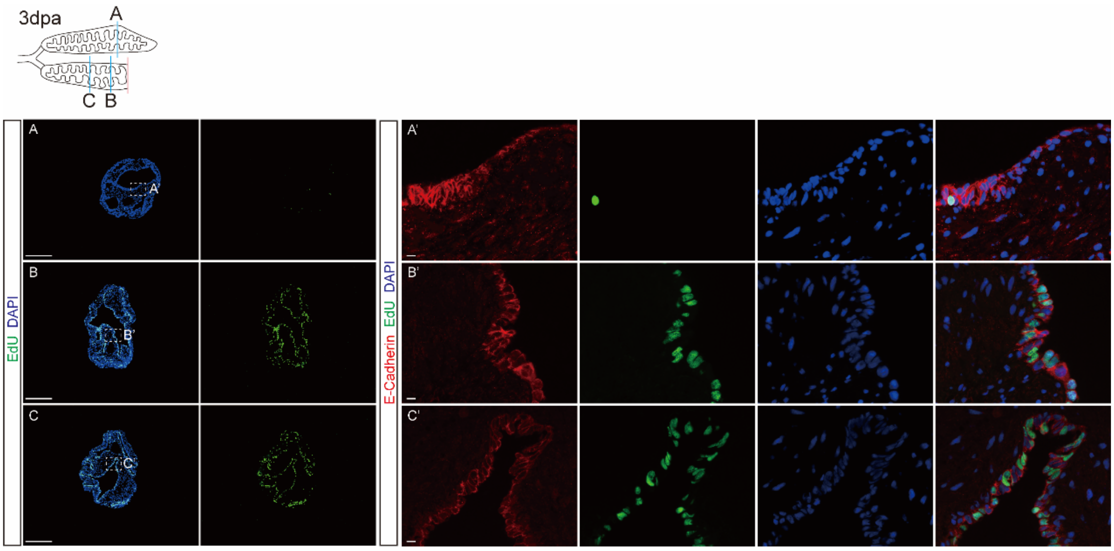

Figure S3. The identification of proliferating epithelial cells on transverse sections of 3dpa lungs in the adult salamanders.

EdU and E-Cadherin co-immunostaining on transverse sections of 3dpa and contralateral uninjured lungs. The red line indicates the damage location, the blue line indicates the sections. A, B, C are the corresponding sections at the blue line, and A', B', C' are the corresponding magnified views of A, B, C. Scale bar: 500 µm (overview) and 20 µm.

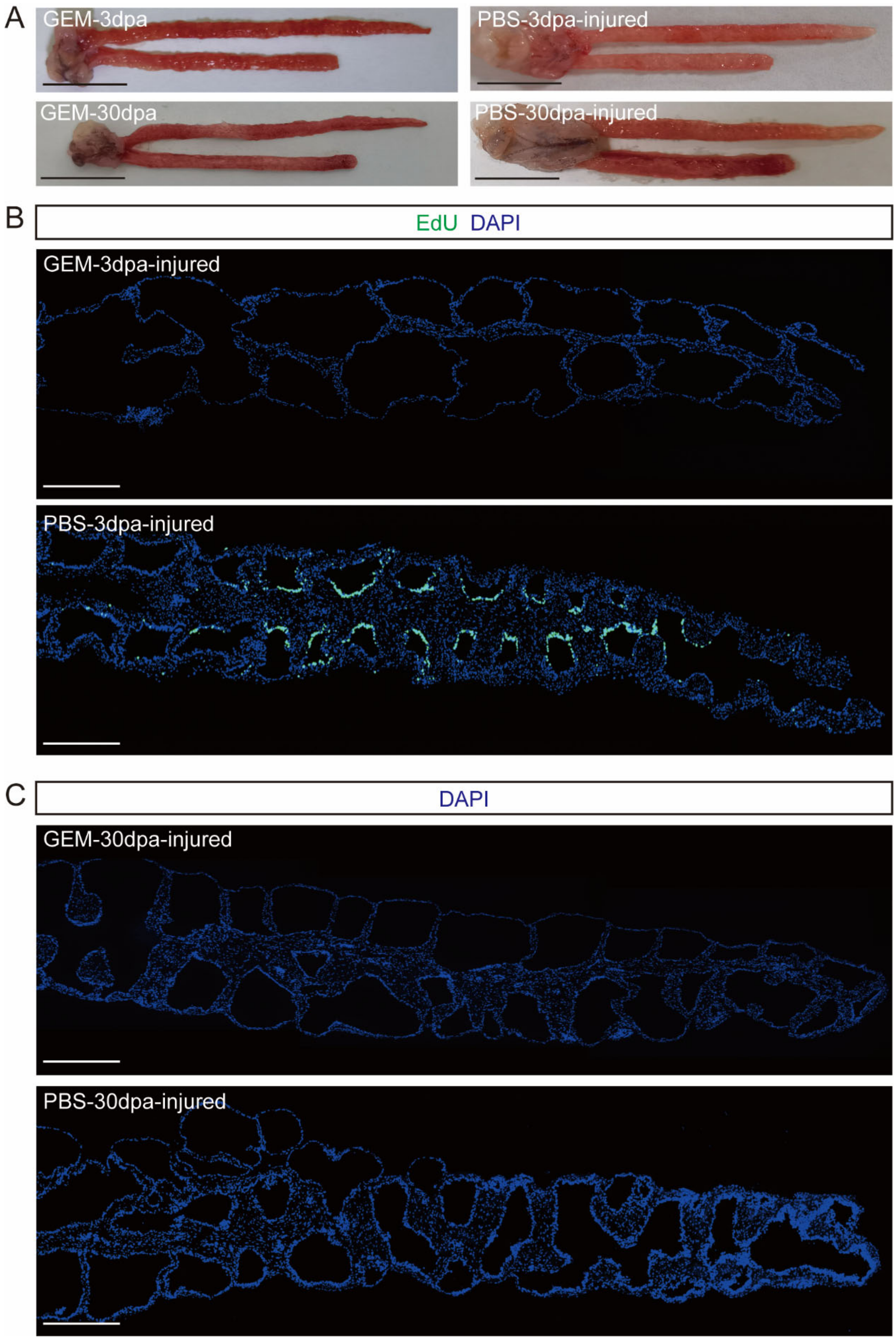

Figure S4. The cell proliferation and lung regeneration were inhibited after the gemcitabine (GEM) treatment in the adult salamanders.

(A) The morphology of the lungs at 3dpa and 30dpa in GEM and PBS-treated animals. n=3 animals. Scale bar: 1cm

(B) The EdU+ proliferating cells in the lungs at 3dpa in GEM and PBS-treated animals. Please note that almost no proliferation was observed in the GEM-treated animals. n=3 animals. Scale bar: 1mm

(C) The lung thickness at 30dpa in GEM and PBS-treated animals. Please note that the thickness of the alveolar wall is much bigger in the PBS-treated control lungs than the GEM-treated lungs, indicating that the epithelial cells proliferation is necessary for the thickening of alveolar wall. n=3 animals. Scale bar: 1mm

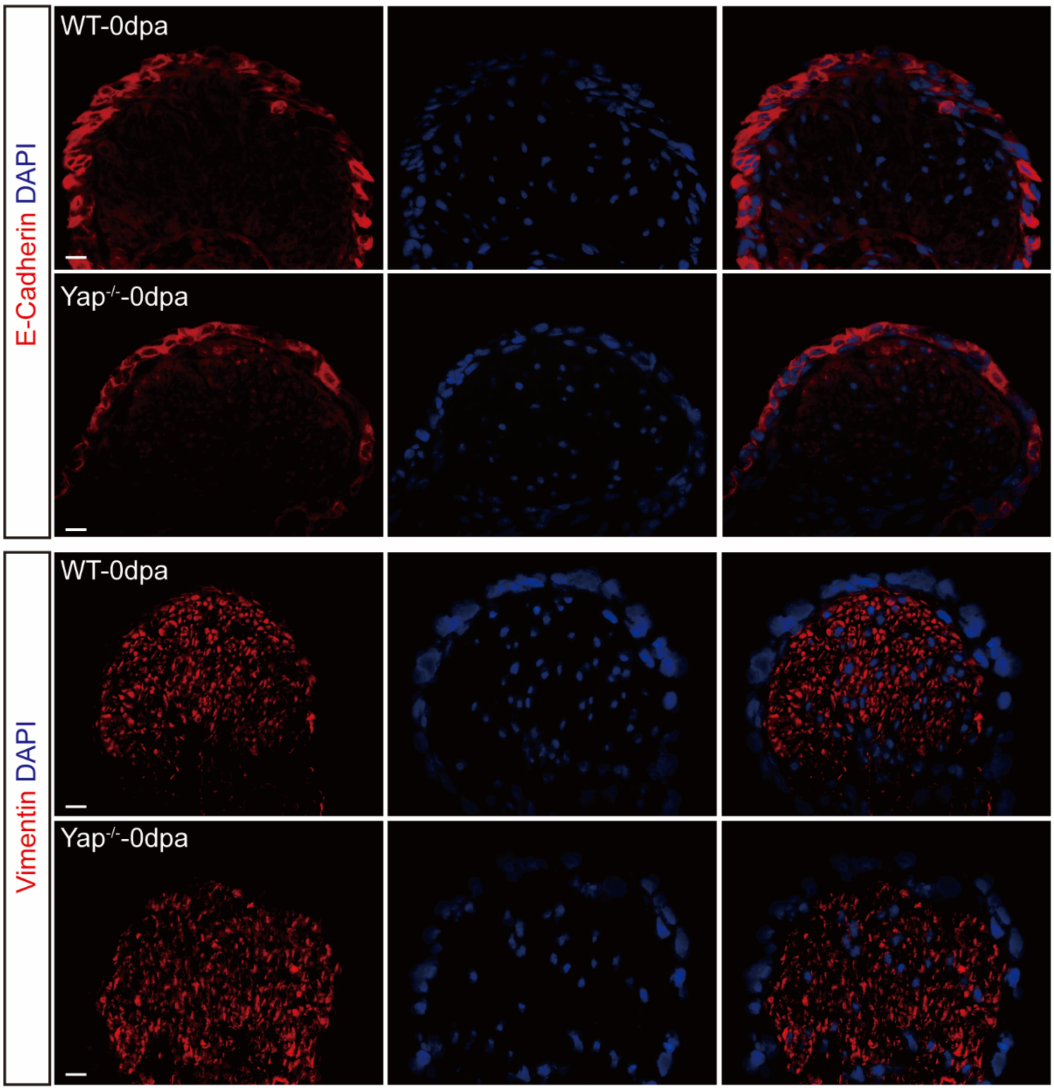

Figure S5. The epithelial cells in WT and Yap mutant animals are similar before injury.

Immunostaining of E-cadherin and Vimentin in adult (one-year-old) WT and $Yap^{-/-}$ animals, respectively. Scale bar: 20 μm.
